# Risk Factors and Clinical Outcomes of Post-Extubation Stridor in Pediatric Intensive Care

**DOI:** 10.3390/children12121698

**Published:** 2025-12-16

**Authors:** Jakeline Godinho Fonseca, Cristiane Fernandes de Moura, Geovana Soffa Rézio, Laís Aparecida da Silva, Mayara Moreira de Deus, Amanda Elis Rodrigues, Juliana Alves de Sousa Caixeta, Luiza Avelino Ferri, Melissa Ameloti Gomes Avelino

**Affiliations:** 1 Programa de Pós-Graduação em Ciências da Saúde, Faculdade de Medicina, Universidade Federal de Goiás (UFG), Goiânia 74690-900, Brazil; crisfono.gyn@gmail.com (C.F.d.M.); geovanasoffa@discente.ufg.br (G.S.R.); mayaramoreiradedeus@gmail.com (M.M.d.D.); amandaelis.fisio@gmail.com (A.E.R.);; 2 Hospital Estadual de Urgências Governador Otávio Lage de Siqueira (HUGOL), Goiânia 74463-350, Brazil; laisprecep@gmail.com; 3 Hospital Estadual da Criança e do Adolescente (HECAD), Goiânia 74860-260, Brazil; 4 Faculdade de Medicina, Faculdade Ciências Médicas da Santa Casa de São Paulo, (FCMSCSP), São Paulo 01224-001, Brazil; luizaavelinoferri06@gmail.com

**Keywords:** respiratory sounds, airway extubation, pediatric intensive care unit, mechanical ventilation

## Abstract

Objectives: To assess risk factors for post-extubation stridor in children and its impact on clinical outcomes. Methods: Prospective cohort study with children aged from 0 to 13 years who were intubated or underwent orotracheal intubation in the pediatric intensive care units (PICU) of two tertiary public hospitals. The outcome of interest was the occurrence of post-extubation stridor. The information collected included patient characteristics, comorbidities, history of airway manipulation, and factors related to orotracheal intubation. A logistic regression was used to identify potential risk factors for post-extubation stridor; data were analyzed until hospital discharge, death, or referral to another facility. Results: A total of 239 children were included, with a median age of 1.3 years and a duration of intubation of three days. Post-extubation stridor was observed in 57.3% of children. A multivariate analysis included prehospital or non-specialized hospital intubation, trauma or complications during intubation, and orotracheal intubation longer than seven days as risk factors for stridor. Children with stridor had a longer PICU length of stay, longer duration of invasive mechanical ventilation, and were often managed with non-invasive ventilation (*p* < 0.05). Most children with extubation failure (*p* = 0.001) and cardiorespiratory arrest (*p* = 0.03) presented with stridor. Conclusions: Risk factors for post-extubation stridor included intubation performed in prehospital or non-specialized hospitals, orotracheal intubation longer than seven days, and trauma or complications during intubation. Children with stridor had a worse prognosis, with longer stays in the PICU and on mechanical ventilation and higher rates of extubation failure.

## 1. Introduction

Orotracheal intubation poses a high risk in critically ill children with difficult airways, unstable hemodynamics, or respiratory failure [1]. Compared with adults, children have distinct airway anatomy, lower functional residual capacity, and higher oxygen demand [2]. Orotracheal intubation can lead to complications, including laryngeal edema and stenosis, granulation tissue, and vocal fold paralysis, which may result in upper airway obstruction after extubation [3].

Upper airway obstruction is the leading cause of extubation failure in children, and the post-extubation stridor is the main sign of this obstruction [3,4,5]. Extubation failure occurs in 3% to 30% of pediatric patients [4,5,6,7,8,9,10] and is associated with prolonged hospital and intensive care unit (ICU) length of stay, longer duration of mechanical ventilation, increased reintubation and airway injury, higher costs, tracheostomy, and greater morbidity and mortality [4,6,11].

The incidence of post-extubation stridor in children ranges from 4.5% to 44.4% [12,13]. Potential risk factors for stridor and laryngeal injuries include previous orotracheal intubation, comorbidities (e.g., gastroesophageal reflux disease and prematurity), traumatic intubation, inappropriate tube size, use of cuffed tubes or high cuff pressures, prolonged intubation, multiple tube repositioning, reintubation, inadequate sedation, and infection [14,15,16,17,18,19,20,21,22].

In this context, identifying risk factors for post-extubation stridor is crucial for implementing best clinical practices in intubated children, reducing complications, optimizing interventions, and preventing severe laryngotracheal stenosis or tracheostomy. Therefore, this study aimed to investigate risk factors for post-extubation stridor in children and its impact on clinical outcomes.

## 2. Materials and Methods

This prospective cohort study was conducted between September 2022 and September 2023 within 60 beds of six pediatric intensive care units (PICU) in two public hospitals in central Brazil. The study was approved by the research ethics committee (CAAE 55640121.4.0000.5082), and written informed consent was obtained from the legal guardians of all children.

Children of both genders, aged from 0 to 13 years, who were either admitted while intubated or underwent orotracheal intubation during hospitalization were included. Exclusion criteria comprised children who had a tracheostomy without an extubation attempt, those who died before extubation, underwent extubation outside the study hospitals, followed a brain death protocol, or received palliative extubation.

Data were collected using a standardized questionnaire, based on previous studies [19,20,22], and refined following a pilot test with nine children. Researchers responsible for data collection were trained in advance. Information was obtained from electronic medical records at both hospitals and from legal guardians. A follow-up was ensured through daily visits to the PICU.

Information was collected on patient characteristics, comorbidities (e.g., syndromes, encephalopathies, neoplasms, congenital heart disease, lung disease, gastroesophageal reflux, prematurity), history of airway manipulation (diagnostic or therapeutic), and intubation-related factors. Researchers also monitored the clinical course, including the use of pre-extubation corticosteroids, post-extubation antibiotics, and inhaled epinephrine.

The outcome of interest was the occurrence of post-extubation stridor within 72 h. The diagnosis was made at the bedside by the clinical care team through auscultation with a stethoscope of the cervical region and thorax to better characterize the presence of the noise. Stridor was classified as present or absent, regardless of severity. Data were analyzed until hospital discharge, death, or transfer to another facility. Endotracheal tube size was considered suitable according to the American Heart Association guidelines (American Heart Association and American Academy of Pediatrics, 2006). Duration of intubation was defined as the interval between intubation and the first extubation. Extubation failure was defined as reinsertion of the endotracheal tube within 72 h after extubation [7].

The sample size calculation assumed that approximately 768 children are intubated annually in the PICU of both hospitals. Based on the literature, the incidence of post-extubation stridor ranges from 4.5% to 44.4% [12,13], and an incidence of 20% was used for calculation. Considering a 95% confidence interval (95% CI) and a 5% margin of error, a target sample size of 187 children was determined.

Data were analyzed using SPSS version 21.0. Continuous variables were reported as medians with 95% CI, while categorical variables as frequencies and percentages. Associations were assessed using the chi-square test (categorical variables) or the Mann–Whitney test (continuous variables). Logistic regression was used to identify potential risk factors for stridor through univariate and multivariate analyses. Statistical significance was set at *p* < 0.05.

## 3. Results

A total of 239 children, with a median age of 1.3 years (0 to 13 years), were included. Stridor occurred in 137 children (57.3%). Table 1 describes the sample characteristics.

Statistically significant differences were observed between children with and without stridor regarding the reason and setting of intubation, the specialty of the professional performing it, intubation-related trauma, and intubation duration exceeding seven days. Table 2 summarizes these results.

A multivariate logistic regression analysis was performed to evaluate the risk factors associated with stridor (Table 3). Variables with *p* < 0.20 in the univariate analysis were included in a subsequent multivariate model (Table 4). Pre-hospital and non-specialized in-hospital intubation, intubation-related injuries or complications, and orotracheal intubation lasting longer than seven days were identified as significant risk factors (*p* < 0.05).

Children with stridor received pre-extubation intravenous corticosteroids more often than those without stridor (81% vs. 59.8%; *p* = 0.001). The same pattern was observed for post-extubation epinephrine nebulization (96.3% vs. 82.4%; *p* = 0.001). No significant differences were found for antibiotic use.

In addition, children with stridor had longer PICU length of stay (median 15.0 vs. 10.5 days; *p* = 0.004) and mechanical ventilation (5.0 vs. 2.0 days; *p* = 0.001) than those without stridor. Extubation failure occurred predominantly in children with stridor (*p* = 0.001), who also often required post-extubation non-invasive ventilation (*p* = 0.001). Most cases of cardiorespiratory arrest (*p* = 0.039) and airway endoscopy (*p* = 0.045) also involved children with stridor. Table 5 summarizes these results.

## 4. Discussion

The risk factors for post-extubation stridor in our sample were intubation performed in prehospital or non-specialized hospitals, orotracheal intubation longer than seven days, and trauma or complications during intubation. Surgery was the primary reason for orotracheal intubation in the total sample, whereas acute respiratory failure was the main reason for children with stridor. This finding corroborates Lambercy et al. [20], who assessed 39 children and reported that most laryngeal injuries occurred after emergency intubations (84.6%), mainly due to clinical reasons (77.0%). Studies report that emergency intubations increase the risk of hypoxia, hypotension, airway injury, and multiple intubation attempts, often due to lack of equipment, incomplete medical history, or the urgent nature of the procedure [16,23,24,25]. Furthermore, trauma or difficulty during intubation is often underreported in medical records. The setting in which the orotracheal intubation procedure is performed may influence the occurrence of stridor. Veder et al. [22] reported that children intubated by prehospital services were five times more likely to develop stridor than those intubated in the hospital, due to emergency and traumatic procedures, often performed by inexperienced professionals. In addition, the duration of orotracheal intubation may be associated with post-extubation stridor. Nascimento et al. [19] reported an association between intubation lasting more than three days and the development of stridor. Intubation exceeding seven days has been linked to more severe airway injuries, assessed by endoscopy, in a sample of 39 children with a mean age of 3.35 years [20], and to subglottic stenosis, with the risk increasing by 50.3% for every additional five days [17,21]. However, a recent systematic review found no association between duration and intubation-related injuries [12].

Our study highlights that over 70% of children were intubated with cuffed endotracheal tubes, which was not a risk factor for stridor. Evidence on the use of cuffed endotracheal tubes is conflicting. Nascimento et al. [19] assessed 136 young children and found no association between cuffed tubes and stridor, whereas Veder et al. [22] reported a higher risk in those younger than one year in a similar sample size. Furthermore, a systematic review found no significant difference in the incidence of stridor between cuffed and uncuffed orotracheal tubes, with a low quality of evidence [26]. Similarly, Chen et al. [27] reported that the presence of a cuff was not associated with stridor; however, uncuffed tubes were associated with a higher need for tube replacement, which has been linked to moderate to severe airway injury in children [28].

Moreover, 57.3% of children had post-extubation stridor, which is higher than previously reported incidences of 4.5% to 44.4% [12,13,19,22]. Part of this difference may be attributed to the complexity and heterogeneity of our sample, as our PICUs are organized into specialized units–trauma, cardiology, neurology, respiratory, and general–which concentrate postoperative patients complex surgical procedures as well as individuals with diverse and severe underlying conditions. Another important factor is that this was a prospective study, in which active and systematic search for stridor was conducted using stethoscope auscultation as the diagnostic method. This implies that respiratory signs which, in other settings or in retrospective studies, might go unnoticed or be underrecognized were identified and recorded in our cohort. Thus, the careful and deliberate detection of even subtle episodes of stridor likely contributed to the higher incidence observed.

Furthermore, children with stridor had a longer ICU length of stay than those without stridor, corroborating Algebaly et al. [14]. In a large retrospective study with 14,045,425 patients under 20 years old in the United States, the hospital length of stay was longer for patients with subglottic stenosis (13.11 vs. 3.76 days), a clinical condition typically associated with stridor (13.11 vs. 3.76 days) [29].

Additionally, children with stridor required longer mechanical ventilation. This finding is consistent with Lambercy et al. [20], who found that moderate to severe laryngeal injuries were associated with prolonged intubation, with most of these patients presenting stridor. Likewise, children with stridor were more likely to require noninvasive ventilation after extubation. In a multicenter study involving 2794 children (mean age 15.5 months), extubation failure was associated with airway rescue interventions, including steroids, epinephrine, heliox, or non-invasive ventilation, within 24 h after extubation [4].

The extubation failure rate in our study is consistent with previous evidence (16.8%) [4,8,9,10]. Stridor was associated with extubation failure, agreeing with Simonassi and Sanso [10], who reported a 5.84-fold increased risk of extubation failure among children with post-extubation stridor. These findings highlight stridor as a clinical marker of laryngeal injury and a warning sign of potential adverse outcomes.

The laryngotracheoscopy is the gold standard for diagnosing acute post-intubation injuries and is recommended in cases of persistent stridor or repeated extubation failures [3,11,13,30]. Early diagnosis of these injuries is essential to guide timely interventions and reduce the need for complex procedures such as tracheostomies and open surgeries [3,15]. However, the reality of Brazilian pediatric ICUs—including the institutions participating in this study—is still marked by the absence of specialized pediatric airway teams and the limited availability of endoscopic equipment. This structural limitation, common in developing countries, reinforces the relevance of identifying simple and accessible clinical markers for early risk stratification. In this context, our study stands out for using post-extubation stridor as a clinical sign potentially predictive of negative outcomes, an assessment that becomes especially important in settings where access to the diagnostic gold standard is limited. The high incidence of stridor observed may reflect both characteristics of the study population and the inherent variability of clinical diagnosis performed by different healthcare professionals; this aspect should be interpreted as part of the real-world environment of ICUs caring for critically ill children in Brazil. Despite limitations related to the absence of endoscopic evaluation and the potential bias of clinical assessment, the study presents important strengths: a prospective design, a large sample, a multicenter character involving six pediatric ICUs, and data collection performed exclusively by three trained researchers, ensuring greater uniformity in the documentation of variables. Additionally, by including patients with different clinical profiles and multiple indications for intubation, we broaden the applicability of the findings to daily practice. Thus, identifying risk factors associated with post-extubation stridor in this study contributes to improving best practices in pediatric extubation, providing evidence applicable to resource-limited contexts and reinforcing the need for careful clinical monitoring during the post-extubation period.

## 5. Conclusions

The risk factors identified in our study for post-extubation stridor included intubations in prehospital settings or non-specialized hospitals, injuries or complications during orotracheal intubation, and orotracheal intubation longer than seven days. Our children with stridor had worse clinical outcomes compared with those without stridor, including longer invasive mechanical ventilation and PICU length of stay and greater use of non-invasive ventilation after extubation. Most children with extubation failure, and most of those who experienced cardiorespiratory arrest or underwent airway endoscopy, presented with stridor.

## Figures and Tables

**Table 1:** Sample characteristics (*n* = 239).

Variable	With Stridor *n* (%)	Without Stridor *n* (%)	Total *n* (%)	*p*
Gender				
Male	78 (56.9%)	60 (58.8%)	138 (57.7%)	0.77
Female	59 (43.1%)	42 (41.2%)	101 (42.3%)	
Age				
>28 days	14 (10.3%)	21(20.8%)	35 (14.8%)	0.022 *
28 days to 2 years	66 (48.5%)	34 (33.7%)	100 (42.2%)	
>2 years	56 (41.2%)	46 (45.5%)	102 (43.0%)	
Prematurity	27 (19.7%)	19 (18.6%)	46 (19.2%)	0.83
Comorbidities	52 (38.0%)	53 (52.0%)	105 (43.9%)	0.03 *
Previous orotracheal intubation	28 (20.4%)	22 (22.0%)	50 (21.1%)	0.77
History of laryngitis	16 (12.0%)	16 (16.3%)	32 (13.9%)	0.35
Upper airway malformation	1 (0.7%)	0 (0.0%)	1 (0.4%)	0.38
Previous upper airway surgery	0 (0.0%)	1 (1.0%)	1 (0.4%)	0.24

Chi-square test; * *p* < 0.05.

**Table 2:** Description of orotracheal intubations (*n* = 239).

Variable	With Stridor *n* (%)	Without Stridor *n* (%)	Total *n* (%)	*p*
Reason for orotracheal intubation				
Surgery	32 (23.4%)	53 (52.0%)	85 (35.6%)	0.001 *
Acute respiratory failure	53 (38.7%)	29 (28.4%)	82 (34.3%)	
Low level of consciousness	39 (28.5%)	16 (15.7%)	55 (23.0%)	
Other reasons	13 (9.5%)	4 (3.9%)	17 (7.1%)	
Setting of orotracheal intubation				
Prehospital	18 (13.1%)	12 (11.8%)	30 (12.6%)	0.029 *
Emergency department	17 (12.4%)	13 (12.7%)	30 (12.6%)	
Pediatric intensive care unit	29 (21.2%)	17 (16.7%)	46 (19.2%)	
Surgical center	23 (16.8%)	35 (34.3%)	58 (24.3%)	
Other hospitals	50 (36.5%)	25 (24.5%)	75 (31.4%)	
Specialty of orotracheal intubation				
Anesthesiologist	31 (22.6%)	48 (47.1%)	79 (33.1%)	0.002 *
Pediatrician	55 (40.1%)	34 (33.3%)	89 (37.2%)	
Other specialties	51 (37.2%)	20 (19.6%)	71 (29.7%)	
Inadequate orotracheal tube	69 (51.1%)	45 (44.6%)	114 (48.3%)	0.319
Tube with cuff	98 (72.6%)	68 (66.7%)	166 (70.0%)	0.324
Intubation-related injuries	21 (40.4%)	6 (12.2%)	27 (26.7%)	0.023 *
Orotracheal intubation exceeding seven days	42 (30.9%)	13 (12.9%)	55 (23.2%)	0.001 *

Chi-square test; * *p* < 0.05.

**Table 3:** Univariate and multivariate analyses of risk factors for stridor (*n* = 239).

Variable	Stridor *n* (%)	Total *n*	Univariate		Multivariate	
			Odds Ratio (95% CI)	*p*	Odds Ratio (95% CI)	*p*
Prematurity						
Yes	27 (58.7%)	46	0.9 (0.5–1.8)	0.834	0.7 (0.3–1.6)	0.422
No	110 (57.0%)	193				
Age						
≤2 years	80 (59.3%)	135	1.2 (0.7–2.1)	0.502	1.5 (0.7–3.0)	0.271
>2 years	56 (54.9%)	102				
Comorbidities						
Yes	52 (49.5%)	105	1.8 (1.0–2.9)	0.032 *	1.5 (0.7–3.1)	0.295
No	85 (63.4%)	134				
Previous orotracheal intubation						
Yes	28 (56.0%)	50	1.1 (0.6–2.1)	0.771	1.0 (0.5–2.3)	0.939
No	109 (58.3%)	187				
History of laryngitis						
Yes	16 (50.0%)	32	1.4 (0.7–3.0)	0.352	1.9 (0.8–4.7)	0.126
No	117 (58.8%)	199				
Setting of orotracheal intubation						
Outside study hospitals	68 (64.8%)	105	1.7 (1.0–2.9)	0.040 *	2.0 (1.0–4.1)	0.054 *
At study hospitals	69 (51.5%)	134				
Specialty of orotracheal intubation						
Others	82 (54.7%)	150	1.3 (0.8–2.3)	0.282	1.3 (0.6–2.7)	0.531
Pediatrician	55 (61.8%)	89				
Tube with cuff						
Yes	98 (59.0%)	166	1.3 (0.8–2.3)	0.325	1.5 (0.8–3.1)	0.231
No	37 (52.1%)	71				
Intubation-related injuries or complications						
Yes	21 (77.8%)	27	2.9 (1.1–7.5)	0.028 *	3.9 (1.3–12.0)	0.016 *
No	116 (54.7%)	212				
Adequate orotracheal tube						
Yes	66 (54.1%)	122	0.8 (0.5–1.29)	0.319	0.8 (0.4–1.4)	0.371
No	69 (60.5%)	114				
Duration of orotracheal intubation						
≤seven days	94 (51.6%)	182	3.0 (1.5–6.0)	0.002 *	4.3 (1.6–11.6)	0.003 *
>seven days	42 (76.4%)	55				

95% CI: 95% confidence interval; * *p* < 0.05.

**Table 4:** Multivariate analyses of risk factors for stridor post-extubation (*n* = 239).

Variable	Stridor *n* (%)	Total	Multivariate	
			Odds Ratio (95% CI)	*p*
Comorbidities				
Yes	52 (49.5%)	105	1.6 (0.9–2.9)	0.102
No	85 (63.4%)	134		
Setting of orotracheal intubation				
Outside study hospitals	68 (64.8%)	105	2.1 (1.1–3.8)	0.019 *
At study hospitals	69 (51.5%)	134		
Intubation-related injuries or complications				
Yes	21 (77.8%)	27	3.5 (1.3–9.6)	0.014 *
No	116 (54.7%)	212		
Duration of orotracheal intubation				
≤seven days	94 (51.6%)	182	3.6 (1.7–7.4)	0.001 *
>seven days	42 (76.4%)	55		

95% CI: 95% confidence interval; * *p* < 0.05.

**Table 5:** Clinical outcomes according to the presence of stridor (*n* = 239).

Variable *n* (%)	with Stridor	Without Stridor	Total	*p*
Extubation failure				
Yes	32 (23.5%)	8 (7.8%)	40 (16.8%)	0.001 *
No	104 (76.5%)	94 (92.2%)	198 (83.2%)	
Time to extubation failure				
≤6 h	12 (37.5%)	0 (0.0%)	12 (30.8%)	0.154
6 to 24 h	5 (15.6%)	2 (28.6%)	7 (17.9%)	
24 to 48 h	5 (15.6%)	3 (42.9%)	8 (20.5%)	
48 to 72 h	10 (31.3%)	2 (28.6%)	12 (30.8%)	
Reason for extubation failure				
Airway obstruction	20 (62.5%)	2 (25.0%)	22 (55.0%)	0.057
Other	12 (37.5%)	6 (75.0%)	18 (45.0%)	
Post-extubation non-invasive ventilation				
Yes	74 (54.4%)	33 (32.7%)	107 (45.1%)	0.001 *
No	62 (45.6%)	68 (67.3%)	130 (54.9%)	
Cardiorespiratory arrest				
Yes	25 (18.2%)	9 (8.8%)	34 (14.2%)	0.039 *
No	112 (81.8%)	93 (91.2%)	205 (85.8%)	
Tracheostomy				
Yes	10 (7.3%)	2 (2.0%)	12 (5.0%)	0.062
No	127 (92.7%)	100 (98.0%)	227 (95.0%)	
Airway endoscopy				
Yes	17 (12.5%)	5 (4.9%)	22 (9.2%)	0.045 *
No	119 (87.5%)	97 (95.1%)	216 (90.8%)	
Airway surgery				
Yes	0 (0.0%)	2 (2.0%)	2 (0.8%)	0.100
No	137 (100%)	100 (98.0%)	237 (99.2%)	
Outcome				
Death	6 (4.4%)	4 (3.9%)	10 (4.2%)	0.977
PICU discharge	128 (93.4%)	96 (94.1%)	224 (93.7%)	
Transfer	3 (2.2%)	2 (2.0%)	5 (2.1%)	

PICU: pediatric intensive care unit; * *p* < 0.05.

## Data Availability

The data presented in this study are available on request from the corresponding author due to ethical reasons.
